# EXPERIENCES OF CARE TRANSITIONS FROM HOSPICE: ADRD FAMILY CAREGIVERS SOCIAL CONTEXTS

**DOI:** 10.1093/geroni/igae098.1798

**Published:** 2024-12-31

**Authors:** Karla Washington, Stephanie Wladkowski, Kathryn Coccia, Marla Berg-Weger, Cara Wallace

**Affiliations:** Washington University in St. Louis, St. Louis, Missouri, United States; Bowling Green State University, College of Health & Human Services, Bowling Green, Ohio, United States; Saint Louis University, St. Louis, Missouri, United States; Saint Louis University, St. Louis, Missouri, United States; Saint Louis University, St. Louis, Missouri, United States

## Abstract

Patients with ADRD are the largest group of Medicare hospice beneficiaries. They are also more likely to experience a live discharge than any other diagnosis, particularly after 6 months of care without presence of an acute event, indicating removal due to decertification. Although expected prognoses have changed, patients still require substantial care and caregivers often struggle to process feelings of abandonment and uncertainty. Increased burden is placed on primary caregivers who may be unprepared for this transition and are more likely to be socially isolated and lonely. This study interviewed caregivers of patients with ADRD who experienced a live discharge from hospice (n=32). Using the ecological model of resilience for caregivers, this presentation examines how caregivers describe their circle of support (CoS), the way their CoS functions (or does not function) when caregivers need help, and the attributes that impact how and when they utilize members in their supportive network. Results suggest that experiences of care transitions are buffered by patient’s location of care (home versus facility) and by availability of resources (both financial and fulfillment of core functions by CoS members). Understanding the way support systems help caregivers during this time of transition provides important implications for assessment during a live discharge to help hospices identify caregivers that may need additional support and resources during the discharge. With a growing population of patients living with ADRD and the rates in which live discharge impacts patients with ADRD-related diagnoses, supporting caregivers during this critical care transition is of utmost importance.

